# Correction to ‘*In vivo* analysis of *Pim-1* deficiency’

**DOI:** 10.1093/nar/gkad728

**Published:** 2023-08-31

*Nucleic Acids Research*, Volume 21, Issue 20, 11 October 1993, Pages 4750–4755, https://doi.org/10.1093/nar/21.20.4750

In the originally published version of this manuscript, there was a typographical error in the first author's last name. This should read: “Laird” instead of: “Larid”.

This error has been corrected in the article.

